# Familial Multiple Trichodiscomas: Case Report and Concise Review

**DOI:** 10.7759/cureus.1596

**Published:** 2017-08-23

**Authors:** Yun Tong, Alvin B Coda, Jeremy A Schneider, Tissa R Hata, Philip R Cohen

**Affiliations:** 1 Department of Dermatology, University of California San Diego; 2 Department of Dermatology, Scripps Health; 3 Department of Dermatology, University of California, San Diego

**Keywords:** irt-hogg-dubé, cancer, discoid, familial, fibromas, multiple, papules, trichodiscoma, folliculin

## Abstract

Familial multiple trichodiscomas is a condition characterized by multiple asymptomatic skin papules. The inheritance pattern has not been established. The skin lesions usually appear in childhood. The diagnosis of the cutaneous papules is established by pathologic evaluation. Birt-Hogg-Dubé syndrome is excluded by not detecting any aberration in the *folliculin* gene locus. Including our patient, 15 index individuals and their families are described. There is no systemic organ involvement or associated malignancies in individuals with this condition.

## Introduction

Familial multiple trichodiscomas (Online Mendelian Inheritance in Man [OMIM] # 190340), also known as familial multiple discoid fibromas, is a genodermatosis characterized by benign whitish-to-flesh-colored papules featured prominently on the face and ears. This condition shares morphological features with Birt-Hogg-Dubé syndrome (OMIM # 135150); however, it is not linked to the *folliculin *(*FLCN*) gene locus and does not demonstrate pulmonary manifestations or associated visceral malignancy [1]. Familial multiple trichodiscomas has not been widely recognized as a separate condition from Birt-Hogg-Dubé syndrome [1]. Fourteen index individuals and their families have been previously reported. We describe a Chinese man with familial multiple trichodiscomas and review the characteristics of the previous patients with this condition.

## Case presentation

A 39-year-old man of Chinese descent without any significant past history presented for an evaluation of skin lesions on his head, neck and trunk. He commented that he had “always” had the papules. Yet, they had increased in size and number in recent years.

A detailed three-generation family history of 15 total members revealed no one with similar papules. However, the patient’s mother was noted to have skin tags during pregnancy, which spontaneously resolved. Both of the patient’s daughters, who are four years old and one year old, are currently asymptomatic.

The patient has no personal or family history of kidney cancers or pneumothoraces; however, a maternal aunt developed a gastrointestinal cancer and died in her 60s.

**Figure 1:**
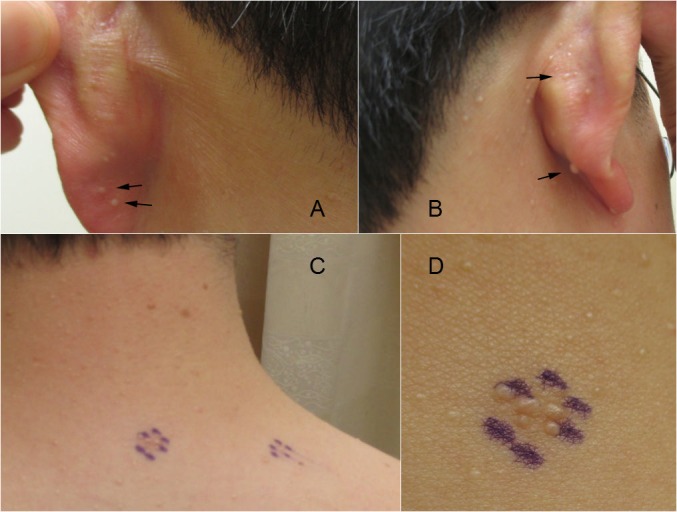
Familial multiple trichodiscoma-associated trichodiscomas. Distant (A, B, C) and closer (D) views of trichodiscomas presenting as numerous 2-4 mm whitish papules on the left (A) and right (B) posterior aspect of the ears (arrows) and the right side of the upper back (lesions present within the areas circled in purple, C and D) of a 39-year-old man.

Cutaneous examination was remarkable for more than 50 pinpoint-to-two millimeter, firm, white-to-flesh-colored papules located on the face, ears, neck, and trunk (Figure 1). Two lesions located on his back were biopsied.

**Figure 2:**
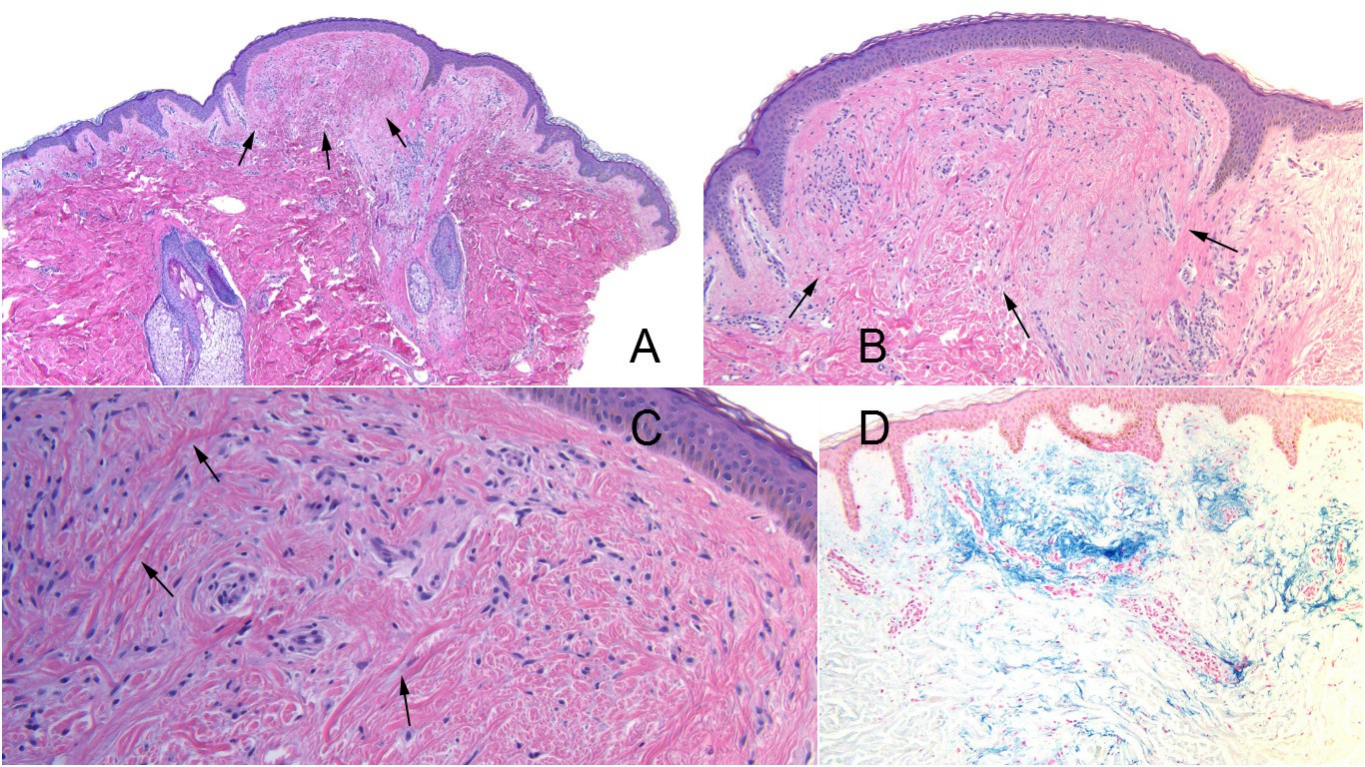
Histopathology of familial multiple trichodiscoma-associated trichodiscoma. A punch biopsy of the trichodiscoma on the right side of the upper back of a 39-year-old man shows a dome-shaped papule in the center of the specimen which extends from an underlying hair follicle with an associated sebaceous gland; the three arrows demarcate the inferior border of the dermal papule (A and B). The overlying epidermis is flattened and the elongated epithelial rete ridges extend around the borders of the dermal tumor (A and B). Collagen deposition is increased around the blood vessels in the dermis (B and C); representative increased collagen fibers are demonstrated by arrows (C). The fibroblasts in the dermis are also increased (C). Mucin is increased in the dermal stroma (B and D); the mucin appears blue after tissue sections are stained with colloidal iron (D) [hematoxylin and eosin: A, x4; B, x10; C, x20; colloidal iron: D, x10].

Microscopic evaluation of both lesions showed similar changes. There were an increased number of interstitial fibroblasts in the dermis. The mucin in the papillary dermis was also increased, which was confirmed by a colloidal iron stain (Figure 2). The pathology changes were consistent with the diagnosis of trichodiscoma.

The patient underwent genetic testing for the aberration of the *folliculin *gene locus. The results were negative for pathogenic mutations, variants of unknown significance, or gross deletions/duplications. Correlation of the clinical morphology and pathology of the skin lesions, the absence of associated systemic manifestations, and the lack of any aberration of the *folliculin *gene locus established the diagnosis of familial multiple trichodiscomas (Table 1) [1].

**Table 1:** Comparison of features in familial multiple trichodiscomas and Birt-Hogg-Dubé syndrome.

	Familial multiple trichodiscomas	Birt-Hogg-Dubé syndrome
Lesion	Trichodiscoma	Fibrofolliculoma, perifollicular fibroma, and trichodiscoma
Morphological feature	1-4 mm papules	1-4 mm papules
Location of skin lesions	Favors face, neck, and trunk; appearance on the ear’s pinna maybe pathognomonic	Favors face, neck, and trunk
Age of onset	Early age, childhood	30s and older
Inheritance	Likely autosomal dominant	Autosomal dominant
Systemic associations	None	Lung cysts and pneumothoraces
Malignancy	None	Yes (renal cancer)
*FLCN* mutation	No	Yes

## Discussion

Trichodiscomas are benign fibrovascular hamartomas that present as small benign papules [2]. In 1977, Birt, Hogg, and Dubé noted trichodiscomas in a triad consisting with the simultaneous occurrence of fibrofolliculomas and acrochordons in a family, suggesting a genodermatosis now known as Birt-Hogg-Dubé syndrome [1]. Most reports of multiple trichodiscomas have been in patients diagnosed with Birt-Hogg-Dubé syndrome. However, a few reports have highlighted patients and family having multiple trichodiscomas without associated malignancies or systemic stigmata of Birt-Hogg-Dubé syndrome [1, 3-8]. These cases, without systemic manifestations or cancer, represent a distinct entity known as familial multiple trichodiscomas.

The incidence of familial multiple trichodiscomas is unknown [1, 6]. It is suspected that many cases prior to the availability of *FLCN *gene testing may have been misdiagnosed as Birt-Hogg-Dubé syndrome. Although believed to be a genodermatosis separate from the *FLCN *gene, the familial multiple trichodiscomas gene has not been identified. While a negative *FLCN *makes Birt-Hogg-Dubé syndrome less likely, the possibility for an undiscovered mutation or variant continues to exist. Indeed, in Birt-Hogg-Dubé syndrome patients, only 91-93% of germline pathogenic mutations can be detected [1-2].

The inheritance pattern of familial multiple trichodiscomas is unsettled. Autosomal-dominant inheritance has been described for a majority of the patients [1]. However, Wee, et al. also suggest an autosomal-recessive pattern of inheritance or germline mosaicism [6].

Familial multiple trichodiscomas have previously been described in 14 European individuals and their families (Table 2) [1, 3-8]. The largest case series come from the same authors who initially suggested familial multiple trichodiscomas was a distinct entity from Birt-Hogg-Dubé syndrome [1]. In contrast to the previously reported individuals with familial multiple trichodiscomas, our patient is of non-European descent. Although none of our patient’s relatives feature similar skin lesions, his high number of lesions and *FLCN*-negative result support our diagnosis of de novo familial multiple trichodiscomas in this individual.

**Table 2:** Characteristics of patients with familial multiple trichodiscomas. F = female; L = limbs; M = male; N = neck; N/A = not available; P = pinna; Pt = patient; Ref = Reference Article(s); T = trunk; UK = United Kingdom; USA = United States of America; * = non-pathogenic *FLCN *variant; ^ = also negative for TSC1 (tuberous sclerosis 1) and TSC2 (tuberous sclerosis 2); ° = also negative for TSC1 (tuberous sclerosis 1), TSC2 (tuberous sclerosis 2), and PTEN (phosphatase and tensin homolog).

Ref.	Individual/family	Sex	Age at lesion onset	Age at presentation	Location of papules	Other lesions	*FLCN*	Malignancy	Systemic	Nationality of Pt	Country of report
Current report	W-C	M	“Always had them”	39	Face, P, N, T	Acrochordons	Negative	Negative	None	Chinese	USA
[7]	Index Pt	M	10 years prior	29	Face, P, L	Negative	Negative	Negative	None	Italian	Italy
	Mother of index Pt	M	Not stated	Not stated	Face	Negative	Not done	Negative	None	Italian	Italy
	Maternal grandfather of index Pt	F	10 years prior	Not stated	Face	Negative	Not done	Negative	None	Italian	Italy
[6]	Index Pt	M	5	27	Face, P, T, L	Negative	Negative	Negative	None	Polish origin	UK
	Sister of index	F	Adolescence	22	Face, L	Negative	Negative	Negative	None	Polish origin	UK
[1]	Index Pt: 11	M	17	70	Face, P	Negative	Positive*	Negative	Renal cysts	Dutch	The Netherlands
	Family of 11	F	22	42	Face, P	Negative	Positive*	Negative	None	Dutch	The Netherlands
	Family of 11	F	15	38	Face, P, L	Negative	Positive*	Negative	None	Dutch	The Netherlands
	Family of 11	M	Not stated	Not stated	Not stated	Not stated	Not stated	Not stated	Not stated	Dutch	The Netherlands
	Index Pt: 17	M	3	34	Face, P	Negative	Negative	Negative	None	Dutch	The Netherlands
	Family of 17	M	1	6	P	Negative	Negative	Negative	None	Dutch	The Netherlands
	Index Pt: 20	F	18	44	Face, P	Negative	Negative	Negative	None	Dutch	The Netherlands
	Family of 20	M	5	14	P	Negative	Negative	Negative	None	Dutch	The Netherlands
	24 – Index Pt	F	Early age	59	Face, P	Negative	Negative	Negative	None	Dutch	The Netherlands
	Family of 24	F	Early age	38	Face, P, L	Negative	Negative	Negative	None	Dutch	The Netherlands
	Family of 24	M	Early age	7	P	Negative	Negative	Negative	None	Dutch	The Netherlands
	Family of 24	M	Early age	7	P	Negative	Negative	Negative	None	Dutch	The Netherlands
	Index Pt: 38	M	25	57	Face, P	Negative	Positive*	Negative	None	Dutch	The Netherlands
	Family of 38	F	18	27	P, T, L	Negative	Positive*	Negative	None	Dutch	The Netherlands
	Family of 38	M	18	28	P	Negative	Positive*	Negative	None	Dutch	The Netherlands
	Family of 38	M	22	24	Face, P, L	Negative	Positive*	Negative	None	Dutch	The Netherlands
	Index Pt: 45	F	18	54	Face, P, T, L	Negative	Positive*	Negative	None	Dutch	The Netherlands
	Family of 45	F	15	51	P	Negative	Positive*	Negative	None	Dutch	The Netherlands
	Family of 45	F	15	48	Face, P, T, L	Negative	Positive*	Negative	None	Dutch	The Netherlands
	Family of 45	F	10	31	P, L	Negative	Positive*	Negative	None	Dutch	The Netherlands
	Family of 45	M	3	24	P	Negative	Positive*	Negative	None	Dutch	The Netherlands
	Index Pt: 50	M	12	73	Face, P	Negative	Negative	Negative	None	Dutch	The Netherlands
	Family of 50	F	10	40	P	Negative	Negative	Negative	None	Dutch	The Netherlands
	Index Pt: H	M	5	40	P	Negative	Not done	Negative	None	Dutch	The Netherlands
	Family of H	F	Early age	17	P	Negative	Not done	Negative	None	Dutch	The Netherlands
[5]	Index Pt: CEG	F	34	37	P, N, T	Acrochordons	N/A	Negative	None	Spanish	Spain
	Sister of CEG	F	40	41	T, L	Negative	N/A	Negative	None	Spanish	Spain
[8]	Index Pt	F	One year prior	40	L	Not stated	N/A	Negative	None	Spanish	Spain
	Sister of index Pt	F	Present for years	54	T, L	Not stated	N/A	Not stated	Not stated	Spanish	Spain
[4]	Index Pt	F	~30 years prior	61	N, T, L	Acrochordons	N/A	Negative	None	Italian or British	Italy or UK
	Sister of index Pt	F	"Developed over several years"	58	T, L	Negative	N/A	Negative	None	Italian or British	Italy or UK
[1, 3]	Index Pt: T	M	1	37	Face, P, T, L	Acrochordons	N/A	Negative	None	Dutch	The Netherlands
	Mother of T	F	At birth	70	Face, P, L	Negative	N/A	Negative	None	Dutch	The Netherlands
	Maternal aunt of T	F	Early age	73	Face	Negative	N/A	Negative	None	Dutch	The Netherlands
	Maternal uncle of T	M	Not stated	Deceased	Not stated	Not stated	N/A	Not stated	Not stated	Dutch	The Netherlands
	Maternal uncle of T	M	Not stated	Deceased	Not stated	Not stated	N/A	Not stated	Not stated	Dutch	The Netherlands
	Maternal aunt of T	F	Not stated	Deceased	Not stated	Not stated	N/A	Not stated	Not stated	Dutch	The Netherlands
	Maternal grandmother of T	F	Not stated	Deceased	Not stated	Not stated	N/A	Not stated	Not stated	Dutch	The Netherlands

A subset of patients (Table 2: families 11, 38, and 45) tested positive for a non-pathogenic *FLCN *variant. It is unclear whether the non-pathogenic mutations are merely coincidental or a milder subtype of Birt-Hogg-Dubé syndrome. The investigators noted that the segregation analysis for two of the families (Table 2: families 11 and 45, which share a common ancestor) determined that involvement of the *FLCN *locus was excluded [1]. Family 38’s mutation was a substitution of an evolutionarily nonconserved amino acid, which lead the researchers to conclude that the mutation was unlikely to have a pathogenic effect and did not cosegregate with the disease [1].

The morphologic features of familial multiple trichodiscomas-associated trichodiscomas are multiple 1-4 mm solid firm dome-shaped, flesh-colored papules. Typically there is a hair at or just outside the periphery of the lesion [3]. Many of the familial multiple trichodiscoma patients reported that their skin lesions appear in childhood; in contrast, the cutaneous lesions of Birt-Hogg-Dubé syndrome appear in middle age [1].

The preferential localization of trichodiscomas on the ear’s pinnae, similar to our patient, may be a clinical feature that is characteristic for familial multiple trichodiscomas. In addition, the trichodiscomas of the ear in patients with familial multiple trichodiscomas may be larger with telangiectasias [1]. Similar to the trichodiscomas of familial multiple trichodiscomas, the fibrofolliculomas of Birt-Hogg-Dubé, usually have a central hair or follicular plug [1].

The pathological changes of a trichodiscoma classically present as a well-defined elliptical or discoid-shaped dermal tumor with flattened overlying epidermis [1]. At the periphery, there is elongated bend of the epidermis with converging rete ridges. The mantles have developed into sebaceous structures with ducts and mucin in trichodiscomas [9]. In contrast to the trichodiscoma of familial multiple trichodiscomas, the trichodiscomas of Birt-Hogg-Dubé syndrome have been shown to have identical anastomosing bands of fibrofolliculomas on deeper sectioning [2].

Fibrofolliculomas, the signature cutaneous feature of Birt-Hogg-Dubé syndrome, are characterized by epithelial strands of basaloid cells extending from the central and hyperplastic hair shaft infundibulum down into the dermis. Trichodiscomas of familial multiple trichodiscomas do not have any features of fibrofolliculomas on deeper sections. In contrast, the trichodiscomas of Birt-Hogg-Dubé syndrome have anastomosing bands, identical to those of fibrofolliculomas, on deeper sectioning. Hence, since the trichodiscomas of familial multiple trichodiscoma do not transition into fibrofolliculomas after deeper sectioning of the biopsy, they represent a distinct pathology separate from that observed in the trichodiscomas of Birt-Hogg-Dubé syndrome [1]. Therefore, in order to avoid further confusion with the trichodiscomas of Birt-Hogg-Dubé syndrome, the original coiners of familial multiple trichodiscomas have attempted to rename the familial multiple trichodiscoma-associated trichodiscomas as discoid fibromas and the disease entity as familial multiple discoid fibromas [1, 6].

Evaluation for an individual in whom the diagnosis of familial multiple trichodiscomas is being considered begins with a detailed history of the onset of their skin lesions, whether there is a family history of similar lesions, and a review of the personal and family history of potentially associated systemic findings such as pneumothoraces or visceral malignancy, such as renal cell carcinoma. Lesion biopsy should be performed; multiple step sections should be evaluated to rule out the trichodiscoma transition to fibrofolliculoma which is observed in Birt-Hogg-Dubé syndrome but is absent in familial multiple trichodiscomas. *FLCN *gene locus testing should be performed; a negative result for a pathogenic gene aberration is consistent with familial multiple trichodiscomas.

As benign lesions, trichodiscomas do not require treatment. Trichodiscomas of familial multiple trichodiscomas are morphologically similar in appearance to the fibrofolliculomas of Birt-Hogg-Dubé syndrome; therefore, similar treatments with lasers or hyfrecation, with or without curettage, may be effective. However, Wee, et al. reported unsatisfactory results after hyfrecation with curettage or shave excision [6].

Topical rapamycin was effective for the treatment of trichodiscomas in a familial multiple trichodiscoma patient; yet, it was not successful for the fibrofolliculomas and trichodiscomas in patients with Birt-Hogg-Dubé syndrome [6, 10]. This observation may provide indirect evidence that the two entities are distinct [6]. However, the improvement with rapamycin also suggests the purported gene involved in familial multiple trichodiscomas may be localized to the mechanistic target of rapamycin (mTOR) pathway, for which Birt-Hogg-Dubé has also been shown to have an intimate relationship [2].

Our review of literature – including the currently described individual – shows 15 families (44 patients) who were diagnosed with familial multiple trichodiscomas (Table 2). Their age of onset is usually younger than that of Birt-Hogg-Dubé syndrome. The lesion location was described in 39 patients: 35 had papules on the head and neck (face, neck, ear’s pinna). If lesions on the pinna are used as a specific finding of familial multiple trichodiscomas, 30 of the 35 patients with descriptions of their trichodiscoma locations were positive. A search for other types of skin lesions was described in 37 of the 44 patients; only four of these individuals also had concurrent acrochordons. Including our patient, evaluation for potentially associated systemic findings was described in 38 patients; only one individual had renal cysts. Likewise, the presence of visceral malignancy was evaluated in 38 patients; none of them had cancer.

## Conclusions

Familial multiple trichodiscomas is a benign condition associated with multiple skin lesions and no systemic manifestations or genomic aberrations. It is characterized by multiple small flesh-colored-to-whitish papules on the head, neck and upper trunk with prominent involvement of the ear’s pinnae. The differential diagnosis of familial multiple trichodiscomas is Birt-Hogg-Dubé syndrome. Although both conditions have cutaneous trichodiscomas, familial multiple trichodiscoma patients do not have condition-associated pulmonary pneumothoraces, visceral cancer, or aberration of the *FLCN *gene locus.
